# Bibliometric analysis of road traffic injuries research in the Gulf Cooperation Council region

**DOI:** 10.12688/f1000research.25903.2

**Published:** 2020-10-16

**Authors:** Farhan Muhammad Butt, Murtaza Ashiq, Shafiq Ur Rehman, Khurram Shahid Minhas, Muhammad Ajmal Khan

**Affiliations:** 1 Transportation and Traffic Engineering Department, Imam Abdulrahman Bin Faisal University, Dammam, Eastern Province, 31441, Saudi Arabia; 2 Library and Information Science, Islamabad Model College for Boys, H-9, Islamabad, Pakistan; 3 Deanship of Library Affairs, Imam Abdulrahman Bin Faisal University, Dammam, Eastern Province, 31441, Saudi Arabia

**Keywords:** road traffic injuries, road traffic accidents, traffic safety, bibliometric analysis, gulf cooperation council

## Abstract

**Background: **Despite governmental interventions, the Gulf Cooperation Council (GCC) region continues to experience higher road traffic crash and fatality rates relative to Western nations. This trend suggests a potential disconnect between Road Traffic Injuries (RTI) research and the mitigation measures put in place.

**Method: **Here, we present an in-depth bibliometric analysis to obtain a comprehensive understanding of RTI research in the GCC region. The Web of Science database was used to search and retrieve the relevant articles during the period of 1981-2019.

**Results: **The volume of RTI research increased from 2015–2019, suggesting an increased focus on traffic safety in the GCC region. Saudi Arabia had the highest RTI research productivity level (126 publications); Bahrain had the lowest (7 publications). Inconsistent with its low publication volume, Hammad Medical Corps of Qatar had the highest citation impact score of 16.33. Global collaboration for RTI research was highest between Saudi Arabia and the United States. The most prevalent publication journal for the region was *Accident Analysis and Prevention*. The most common keywords were “ *road traffic accidents*” and “ *road traffic injuries*”; terms such as “ *mobile phones*”, “ *pedestrian safety*”, “ *pedestrians*”, and “ *distracted driving*” were least common. In the five most productive GCC nations with respect to RTI research (Saudi Arabia, United Arab Emirates, Qatar, Kuwait, and Oman), researchers tended to publish works related to road traffic safety in traffic safety-oriented journals.

**Conclusions:** The quantity and quality of RTI publications in GCC is insufficient to meet the increasing related public health and economic burden in the region. The trends among publication volumes, citations, and impact were inconsistent. There is a lack of research collaboration among the institutions. Most of the research related to RTI is being conducted by researchers with a medical background. Research focusing on pedestrians, cyclists and road user behavior is also inadequate.

## Introduction

**Figure 1.**
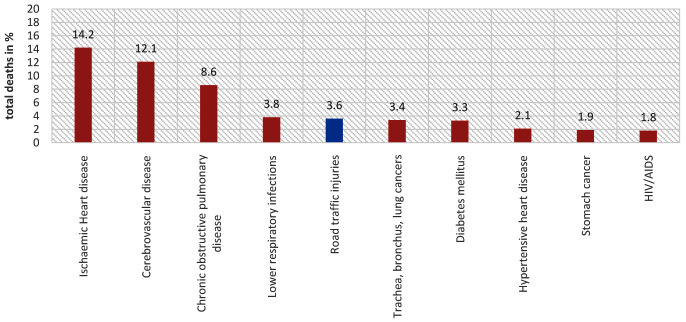
Leading Causes of death in 2030 predicted by WHO ^2^. (generated using Microsoft Excel).

Road traffic injuries (RTI) account for 30% of all deaths worldwide and are the major cause of death among people 15–29 years old ^1^. Figure 1 depicts the leading causes of death expected in 2030 by the World Health Organization (WHO) ^2^. RTI was ranked as the eighth contributor to the total global deaths in 2004; by 2030, RTI is expected to rank as the fifth highest contributor ^2, 3^

The Gulf Cooperation Council (GCC) region (Saudi Arabia, Oman, the United Arab Emirates, Kuwait, Qatar, and Bahrain) experiences substantially higher road traffic crash and fatality rates relative to Western nations. Across 15 developed European nations, the recorded road traffic fatality rate has decreased from 13.5 deaths per 100,000 populations in the 1980s to 5.5 deaths per 100,000 populations today. In contrast, the road traffic fatality rate in the GCC region has remained relatively constant at 23 deaths per 100,000 population over this same period ^4^.

Table 1 compares the 2013 road traffic fatality rates per 100,000 population and per 100,000 registered vehicles for each of the GCC nations, as well as the United Kingdom and the United States ^5^. In the GCC region, Saudi Arabia and Oman had the highest fatality rates per 100,000 population, followed by Kuwait, Qatar, the United Arab Emirates (UAE), and Bahrain ^5^. The population-based fatality rate in the UAE (10.9 deaths per 100,000 population) was comparable to that in the United States (10.6 deaths per 100,000 population). However, the fatality rate based on vehicle ownership in the UAE (38.2 deaths per 100,000 registered vehicles) was significantly higher than that in the United States (12.9 deaths per 100,000 registered vehicles). The United Kingdom had the lowest fatality rates in terms of both population (2.9 deaths per 100,000 population) and vehicle ownership (5.1 deaths per 100,000 registered vehicles) ^5^.

**Table 1.** Comparative road traffic fatality rates of nations reported in WHO Global Status Report on Road Safety 2015 ^5^.

Nation	Deaths/100,000 population	Registered vehicles	Deaths/100,000 registered vehicles
Bahrain	8.0	545,155	19.6
Kuwait	18.7	1,841,416	34.2
Oman	25.4	1,082,996	85.3
Qatar	15.2	647,878	50.9
Saudi Arabia	27.4	6,599,2161	119.7
United Arab Emirates	10.9	2,674,894	38.2
United Kingdom	2.9	35,582,650	5.1
United States	10.6	265,043,362	12.9

As demonstrated in other nations, road traffic crashes and fatalities can be mitigated through a focused program of RTI research that identifies and supports implementation of strategies designed to reduce the magnitude and severity of road traffic crashes and fatalities. Historical RTI research has been conducted in Western nations with a focus on relating aggressive driving behavior and offences to traffic crashes ^6– 8^. Early studies showed that more than 75% of all crashes were caused by driver behavior ^6– 11^. Although driver behavior clearly constitutes an important risk factor, researchers today caution against trying to identify a single crash cause. Socioeconomic and demographic factors, such as age, sex, marital status, education, training, experience, way of life, emotional status, fatigue, reaction time, vision, vigilance, and driving speed may also affect crash occurrence and should be considered as risk factors in RTI research ^12^.

The high and sustained number of road traffic crashes and fatalities in the GCC region relative to other comparable Western nations poses serious public health and economic challenges and has been well documented in previous studies ^13– 15^. These challenges are exacerbated by the GCC region’s rapidly expanding roadway network and increasing vehicle ownership levels. Unlike demonstrated efforts in Western nations to reduce the magnitude and severity of road traffic crashes and fatalities based on a focused program of research, the high and sustained number of road traffic crashes and fatalities in the GCC region suggests a potential disconnect between local RTI research and any mitigation measures put in place.

To address this potential disconnect, we performed a bibliometric analysis of RTI research in the GCC region using publication data from the Web of Science indexing and abstracting database for 1981–2019. We considered research productivity, institutional and individual authorship, bibliographic coupling and global collaboration, publication journals and publications, and keywords and associated topical trends for the GCC region. In addition, we performed a three-factor analysis using GCC nation, publication journal, and keyword parameters to obtain a more comprehensive understanding of RTI research in the GCC region. The results from this study can inform governmental authorities in the GCC region of research investment needs and academic researchers of research deficiencies and impactful publishing venues. An informed and collective focus on RTI research in the GCC region will help to ensure broader public safety.

## Methods

**Figure 2.**
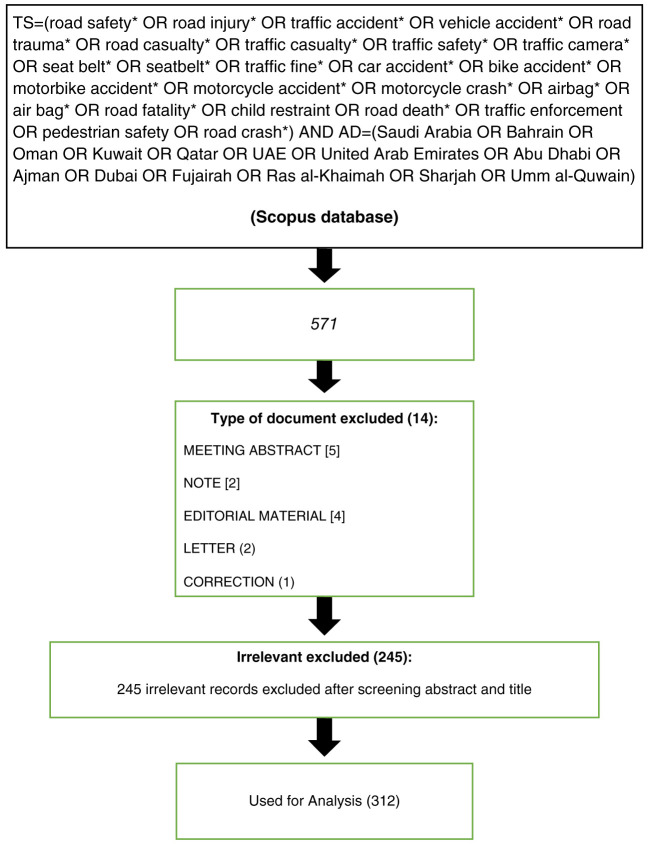
Flow Diagram for Literature Searching Process.

In this study, we performed a bibliometric analysis of RTI research in the GCC region. Bibliometric analysis is a statistical method that collects, analyzes, and extracts various metrics related to research productivity, institutional and individual authorship, publication journals, publications, keywords, and more from published literature. We used data from the Web of Science (WOS) indexing and abstracting database. The WOS database was chosen to extract the relevant data on RTI. Web of Science is the most authentic and reliable indexing and abstracting global database of scholarly literature. Additionally, it also provides full bibliometric data with a simple extraction process, which is suitable for comprehensive bibliometric analysis. The following search query was used in the topic field of the advance search option of the Web of Science Core collection database on November 13, 2019.To get maximum relevant records we used all keywords related to RTI. We searched using the following search terms in the topic field (that is title, keywords and abstract) of the WOS database. These search terms were selected based on literature review and author keyword analysis in different databases. The authors tried to include all search terms for retrieval of entire spectrum of the literature related to study objectives (see Figure 2).

This initial query returned 571 publications. The authors did not apply any filter related to languages and time period to achieve a comprehensive view of RTI research. We refined this initial query by excluding editorial materials (4), notes (2), meeting abstracts (5), letters (2), or corrections (1) as they are not peer reviewed. Research articles, conference papers, review papers, book chapters, and books were included in this study. The complete bibliographic information (title, abstract, author, date of publication, source of publication, keywords, citation count etc.), of the remaining publication records were exported to MS Excel (Microsoft 365) for scrutinizing of these records. Two authors read the title and abstracts of all the records. The third author again repeated this process to ensure the relevancy and reliability of the data. This practice helped the authors to discard 245 irrelevant records. Finally, a total of 312 records were selected for data analysis. The final dataset for this study included 312 publication records comprising 230 journal articles and 82 conference papers ^16^. Data analysis was performed using a combination of spreadsheet and bibliometric analysis software packages such as VOSViewer (version 1.6.15) and Biblioshiny (R Package). The author keyword analysis and bibliographic coupling was done with the help of VOSViewer. Global collaboration, topical trends and three factor analysis were generated by using Biblioshiny.

### Limitations of Study

In this study, we performed a bibliometric analysis of RTI research in the GCC region using data from the Web of Science indexing and abstracting database for 1981–2019. This data was not independently verified to determine if each of the publication records considered for analysis originated from the GCC region. Other databases such as Scopus, PubMed, or Google Scholar may produce a different set of publication records using similar search criteria. Although this comparison was beyond the scope of this study, future work may attempt to verify this study’s findings using data from these alternate sources.

## Results

### Research productivity

**Figure 3.**
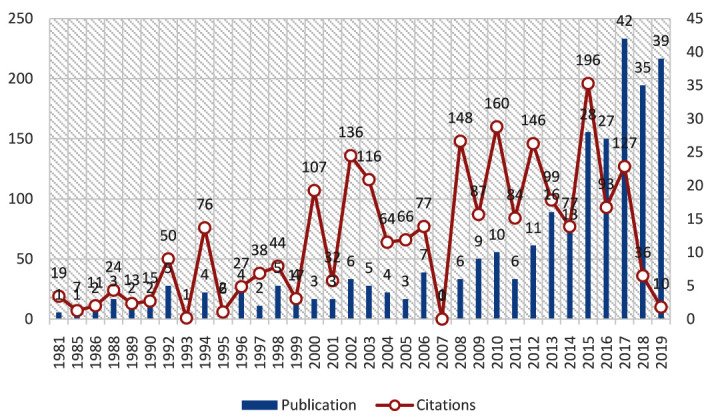
Annual road traffic injuries research productivity in terms of publications and citations in the Gulf Cooperation Council region (1981–2019). (generated using Microsoft Excel).

Figure 3 depicts the annual RTI research productivity in terms of publications and citations in the GCC region. The earliest RTI research publication in the GCC region appeared in 1981 and was cited 19 times. Following that first publication, RTI research productivity gradually increased but remained relatively low. The volume of RTI research substantially increased in 2015–2019, suggesting an increased recent focus on traffic safety in the GCC region. In 2015, 28 publications were cited 196 times. The maximum number of publications (42) occurred in 2017.

Table 2 summarizes RTI research productivity in terms of publications, citations, and citation impact for each nation in the GCC region for 1981–2019. The GCC nations with the highest number of publications included Saudi Arabia (126 publications), the UAE (84 publications), and Qatar (50 publications). Bahrain had only 7 publications.

**Table 2.** Road traffic injuries research productivity in terms of publications, citations, and impact for each nation in the Gulf Cooperation Council region (1981–2019).

Nation	Total publications	Total citations	Citation impact
Saudi Arabia	126	890	7.06
United Arab Emirates	84	654	7.79
Qatar	50	388	7.76
Kuwait	35	182	5.20
Oman	19	80	4.21
Bahrain	7	40	5.71

Publications originating in Saudi Arabia were cited 890 times and had a citation impact of 7.06. Despite lower publication volumes, publications originating in the UAE and Qatar had higher citation impact (7.79 and 7.76, respectively).

### Institutional authorship

Table 3 lists the most productive institutions for RTI research in the GCC region. Four institutions were in Saudi Arabia, three were in the UAE, two were in Qatar, and one was in Kuwait.

**Table 3.** Most productive institutions for road traffic injuries research in the Gulf Cooperation Council region (1981–2019).

Institution	Nation	Total publications	Total citations	Citation impact
United Arab Emirates University	United Arab Emirates	40	545	13.63
King Saud University	Saudi Arabia	37	422	11.41
Qatar University	Qatar	29	105	3.62
Kuwait University	Kuwait	21	141	6.71
Hamad Medical Corporation	Qatar	15	245	16.33
Al Ain Hospital	United Arab Emirates	12	175	14.58
King Saud bin Abdulaziz University of Health Sciences	Saudi Arabia	12	107	8.92
King Abdulaziz University	Saudi Arabia	10	30	3.00
King Khalid University	Saudi Arabia	9	50	5.56
University of Sharjah	United Arab Emirates	8	13	1.63

The UAE University had the highest RTI research productivity with 40 publications cited 545 times with a citation impact of 13.64. King Saud University and Qatar University also had a high number of publications (37 and 29 publications, respectively). The Hamad Medical Corporation and Al Ain Hospital had just 15 and 12 publications, respectively, but their citation impact ranked highest (16.33 and 14.58, respectively).

### Individual authorship

Table 4 lists the most productive individual authors for RTI research in the GCC region. Only three authors had 10 or more publications: F.M.Abu-Zidan (17 publications), K. Shaaban (15 publications), and H.O. Eid (10 publications).

**Table 4.** Most productive individual authors for road traffic injuries research in the Gulf Cooperation Council region (1981–2019).

Individual author	Affiliation	Total publications	Total citations	Citation impact
Abu-Zidan, Fikri M.	UAE University	17	313	18.41
Shaaban, Khaled	Qatar University	15	68	4.53
Eid, Hani O.	Mediclinic Middle East, Dubai	10	170	17.00
Alhajyaseen, Wael K. M.	Qatar University	9	38	4.22
Bendak, Salaheddine	University of Sharjah	8	122	15.25
Hefny, Ashraf F.	UAE University	7	168	24.00
Al-Rukaibi, Fahad	Kuwait University	7	7	1.00
Al-Ghamdi, AS	King Saud University	7	131	18.71
Hassan, Hany M.	University of Central Florida	6	34	5.67
Grivna, Michal	UAE University	6	137	22.83

The 17 publications by F.M. Abu-Zidan were cited 313 times with a citation impact of 18.41. Select authors such as K. Shaaban had a relatively high publication volume (15 publications) but a low number of citations (68 citations) and a low citation impact (4.53). Conversely, A.F. Hefny and M. Grivna had relatively low publication volumes (7 and 6 publications, respectively) but high numbers of citations (168 and 137 citations, respectively) and high citation impact (24.00 and 22.83, respectively).

### Bibliographic coupling among nations

**Figure 4.**
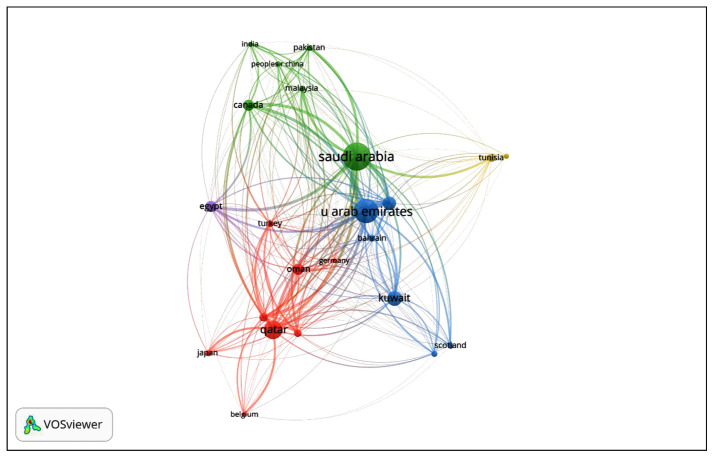
Bibliographic coupling among nations with three or more publications. (generated using VOSViewer software).

Bibliographic coupling is a measure of subject matter commonality among different publications and occurs when two publications reference a common third publication. Figure 4 depicts the bibliographic coupling among different nations. The circle size and colors denote bibliographic coupling levels and different coupling clusters, respectively.

Nations with three or more publications were included; 23 of 45 nations met this criterion. Nations with the highest bibliographic coupling activity included Saudi Arabia (110 documents, 774 citations, and a 5,197 total link strength); the UAE (83 documents, 655 citations, and a 3,731 total link strength); and Qatar (50 documents, 385 citations, and a 3,290 total link strength).

Publications that are closely related in content can be classified into clusters; the connections between the clusters can be described using quantitative network indicators. A visualization of the clustering network can then be generated based on research publication characteristics. Publications spanning different time periods can be clustered and any sub clusters can be merged into a unified visual view ^17, 18^. In this study, nations with three or more publications were included; 23 of 45 nations met this criterion. Bibliographic couplings in the RTI research publications originating in each of these 23 countries were classified into five clusters and visually displayed using the VOSViewer software package.

### Global collaboration among nations

**Figure 5.**
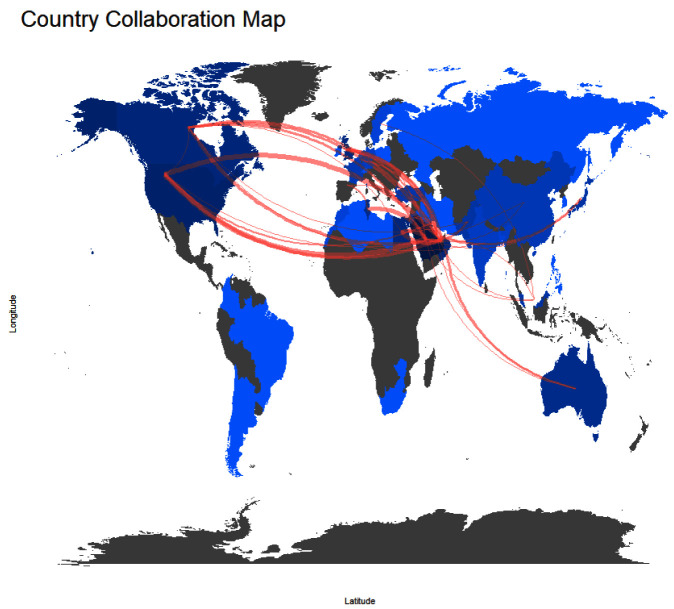
Global collaboration in road traffic injuries research among nations. (generated using Biblioshiny Software R Package).

Figure 5 depicts the historic global collaboration in RTI research among nations. In total, 232 instances (i.e., publications) of global collaboration involving GCC nations were identified. These instances ranged from a single collaboration between two nations to more than 11 repeated collaborations between two nations. The highest levels of collaboration have occurred among Saudi Arabia and the United States (11 instances), Qatar and the United States (9 instances), Egypt and the UAE (8 instances), Saudi Arabia and Tunisia (8 instances), and Qatar and the United Kingdom (7 instances).

### Authorship patterns

**Figure 6.**
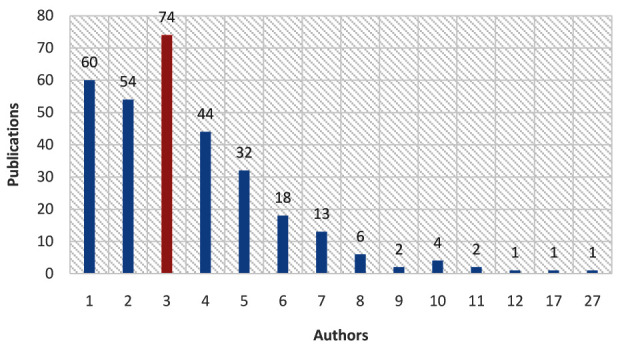
Number of Authors in RTI publications in the GCC region (1981–2019). (generated using Microsoft Excel).

As a supplemental investigation, we considered the number of individual authors on each RTI research publication. Figure 6 shows the number of publications as a function of the number of authors for publications originating in the GCC region. Select publications had more than 10 individual authors, although this was relatively infrequent. Most RTI research publications originating in the GCC region had no more than five authors; the highest number of publications (74) had three authors.

### Publication journals

Table 5 lists the most prevalent publication journals for RTI research in the GCC region. Only five journals had 10 or more publications: Accident Analysis and Prevention (25 publications), Saudi Medical Journal (20 publications), International Journal of Injury Control and Safety Promotion (15 publications), Traffic Injury Prevention (12 publications), and Annals of Saudi Medicine (10 publications). Accident Analysis and Prevention also had the highest number of citations (533) and citation impact (21.32) among these journals.

**Table 5.** Most prevalent publication journals for road traffic injuries research in the Gulf Cooperation Council region (1981–2019).

Publication journals	Total publications	Total citations	Citation impact
Accident Analysis and Prevention	25	533	21.32
Saudi Medical Journal	20	171	8.55
International Journal of Injury Control and Safety Promotion	15	43	2.87
Traffic Injury Prevention	12	91	7.58
Annals of Saudi Medicine	10	93	9.30
Transportation Research Record	7	37	5.29
Journal of The Royal Society of Health	6	62	10.33
Safety Science	6	56	9.33
Transportation Research Part F- Traffic Psychology and Behavior	6	39	6.50
Accident Analysis and Prevention	25	533	21.32

### Highly cited publications

Table 6 lists the 20 most commonly cited RTI research publications originating in the GCC region. Four publications have been cited more than 50 times: “Causes and Effects of Road Traffic Accidents in Saudi Arabia” ^19^ (95 citations); “The Driver Behavior Questionnaire in Arab Gulf Countries: Qatar and United Arab Emirates” ^20^ (60 citations); “Pedestrian-vehicle Crashes and Analytical Techniques for Stratified Contingency Tables” ^21^ (58 citations); and Seat belt Utilization in Saudi Arabia and its Impact on Road accident Injuries” ^22^ (52 citations). Eight of the most cited RTI research publications appeared in Accident Analysis and Prevention.

**Table 6.** Twenty most cited articles with citations on road traffic injuries in Gulf Cooperation Council.

AUTHOR	Title	Source title	Year	Total Citations
Ansari, S; Akhdar, F; Mandoorah, M; Moutaery, K	Causes and effects of road traffic accidents in Saudi Arabia	PUBLIC HEALTH	2000	95
Bener, Abdulbari; Ozkan, Turker; Lajunen, Timo	The driver behaviour questionnaire in Arab Gulf countries: Qatar and United Arab Emirates	ACCIDENT ANALYSIS AND PREVENTION	2008	60
Al-Ghamdi, AS	Pedestrian-vehicle crashes and analytical techniques for stratified contingency tables	ACCIDENT ANALYSIS AND PREVENTION	2002	58
Bendak, S	Seat belt utilization in Saudi Arabia and its impact on road accident injuries	ACCIDENT ANALYSIS AND PREVENTION	2005	52
SHANKS, NJ; ANSARI, M; ALKALAI, D	Road Traffic Accidents in Saudi-Arabia	PUBLIC HEALTH	1994	49
Eid, Hani O.; Barss, Peter; Adam, Shehabeldin H.; Torab, Fawaz Chikh; Lunsjo, Karl; Grivna, Michal; Abu-Zidan, Fikri M.	Factors affecting anatomical region of injury, severity, and mortality for road trauma in a high-income developing country: Lessons for prevention	INJURY-INTERNATIONAL JOURNAL OF THE CARE OF THE INJURED	2009	47
Abbas, Alaa K.; Hefny, Ashraf F.; Abu-Zidan, Fikri M.	Seatbelts and road traffic collision injuries	WORLD JOURNAL OF EMERGENCY SURGERY	2011	44
El-Sadig, M; Norman, JN; Lloyd, OL; Romilly, P; Bener, A	Road traffic accidents in the United Arab Emirates: trends of morbidity and mortality during 1977-1998	ACCIDENT ANALYSIS AND PREVENTION	2002	42
Mansuri, Farah A.; Al-Zalabani, Abdulmohsen H.; Zalat, Marwa M.; Qabshawi, Reem I.	Road safety and road traffic accidents in Saudi Arabia A systematic review of existing evidence	SAUDI MEDICAL JOURNAL	2015	42
Abu-Zidan, Fikri M.; Abbas, Alaa K.; Hefny, Ashraf F.; Eid, Hani O.; Grivna, Michal	Effects of Seat Belt Usage on Injury Pattern and Outcome of Vehicle Occupants After Road Traffic Collisions: Prospective Study	WORLD JOURNAL OF SURGERY	2012	41
Al-Ghamdi, AS	Analysis of traffic accidents at urban intersections in Riyadh	ACCIDENT ANALYSIS AND PREVENTION	2003	41
Bener, A; Abu-Zidan, FM; Bensiali, AK; Al-Mulla, AA; Jaddan, KS	Strategy to improve road safety in developing countries	SAUDI MEDICAL JOURNAL	2003	39
Abbas, KA	Traffic safety assessment and development of predictive models for accidents on rural roads in Egypt	ACCIDENT ANALYSIS AND PREVENTION	2004	38
Bener, Abdulbari; Omar, Azhar O. Kh.; Ahmad, Amal E.; Al-Mulla, Fatma H.; Rahman, Yassir S. Abdul	The pattern of traumatic brain injuries: A country undergoing rapid development	BRAIN INJURY	2010	36
Bener, A; Al-Salman, KM; Pugh, RNH	Injury mortality and morbidity among children in the United Arab Emirates	EUROPEAN JOURNAL OF EPIDEMIOLOGY	1998	36
Nofal, FH; Saeed, AAW	Seasonal variation and weather effects on road traffic accidents in Riyadh City	PUBLIC HEALTH	1997	33
Al-Naami, Mohammed Y.; Arafah, Maria A.; Al- Ibrahim, Fatimah S.	Trauma care systems in Saudi Arabia: an agenda for action	ANNALS OF SAUDI MEDICINE	2010	33
Koushki, PA; Bustan, MA; Kartam, N	Impact of safety belt use on road accident injury and injury type in Kuwait	ACCIDENT ANALYSIS AND PREVENTION	2003	31
Al-Shammari, Naif; Bendak, Salaheddine; Al-Gadhi, Saad	In-Depth Analysis of Pedestrian Crashes in Riyadh	TRAFFIC INJURY PREVENTION	2009	30
Abbas, Alaa K.; Hefny, Ashraf F.; Abu-Zidan, Fikri M.	Does wearing helmets reduce motorcycle-related death? A global evaluation	ACCIDENT ANALYSIS AND PREVENTION	2008	30

**Figure 7.**
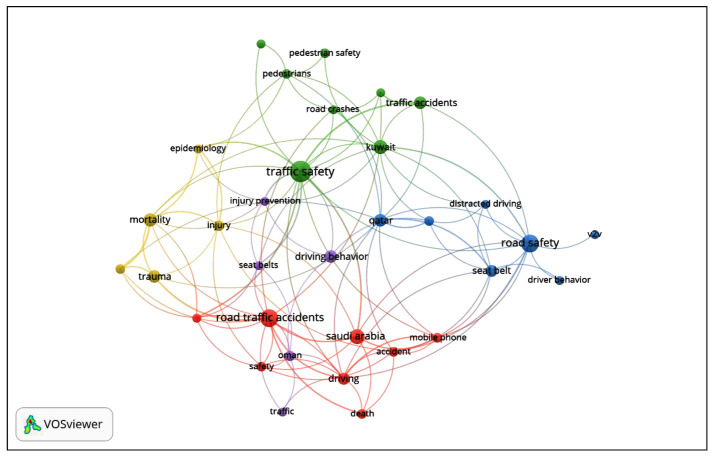
Most common keywords listed four or more times by authors in road traffic injuries research publications originating in the Gulf Cooperation Council region. (generated using VOSViewer software).

Figure 7 depicts the most common keywords listed by authors in RTI research publications originating in the GCC region. The circle size and colors denote keyword occurrence levels and different coupling clusters, respectively.

Keywords that were listed four or more times were included; 33 keywords met this criterion. The most common keywords in RTI research publications were traffic safety (24 occurrences), road traffic injuries (17 occurrences), and road safety (17 occurrences). Terms such as pedestrian safety (5 occurrences), pedestrian (5 occurrences), mobile phone (5 occurrences), driver behavior (4 occurrences), in- jury prevention (4 occurrences), and distracted driving (4 occurrences) were least common.

### Topical trends

**Figure 8.**
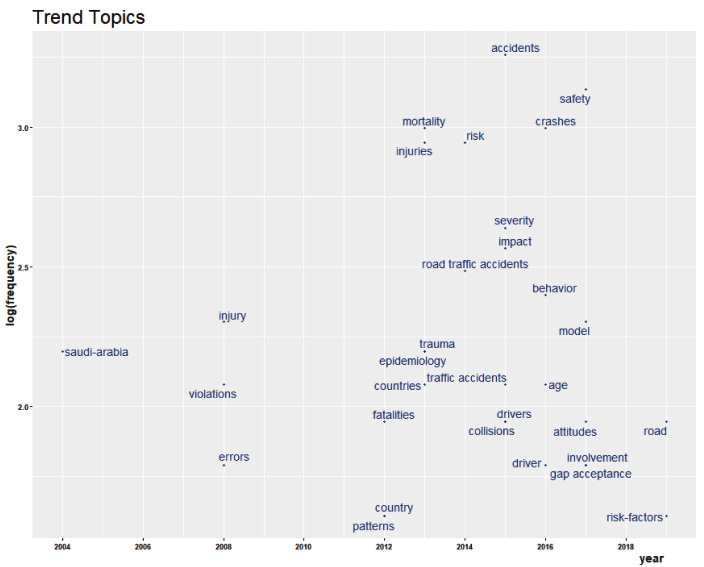
Topical trends in road traffic injuries research in the Gulf Cooperation Council region (1992–2018) based on the five most common keywords per year appearing five or more times. (generated using Biblioshiny Software R Package).

Supplemental to the keyword analysis results presented previously, we considered topical trends in RTI research publications originating in the GCC region from 1992 to 2018. Figure 8 depicts these results. In 1992–2007, RTI research publications were limited to just six topics: Saudi Arabia, hospital, Riyadh, region, accident, and mortality. In 2008, the breadth of topics began to increase. In 2009–2019, the most common RTI research publication topics included road, traffic, safety, and accidents.

### Three-factor analysis using nation, journal, and keyword parameters

**Figure 9.**
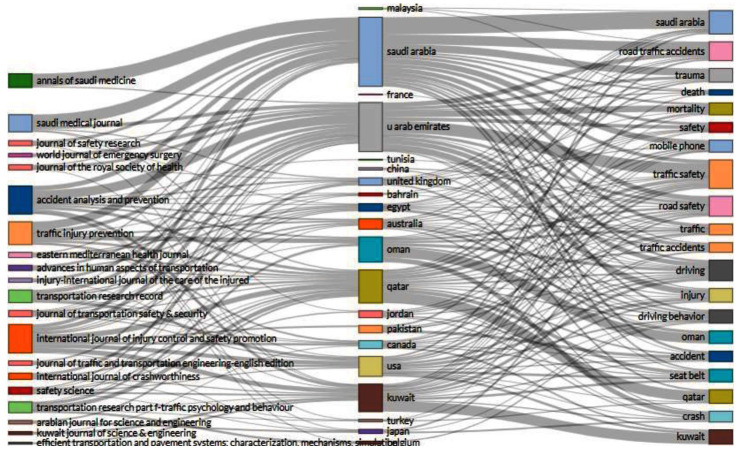
Three-factor analysis of road traffic injuries research in the Gulf Cooperation Council region using nation (center), journal (left), and keyword (right) parameters. (generated using Biblioshiny Software R Package).

As a final step in this study, we performed a three-factor analysis using GCC nation, publication journal, and keyword parameters to obtain a more comprehensive understanding of RTI research in the GCC region. Figure 9 shows the diagram of published literature on RTI Gulf focusing on the relationship among three factors including top keywords, journals and country. Figure 9 presents the results from this analysis.

RTI research originating in the five most productive GCC nations (Saudi Arabia, the UAE, Qatar, Kuwait, and Oman) was most commonly published in just four journals: the *International Journal of Injury Control and Safety Promotion, Accident Analysis and Prevention, Traffic Injury Prevention*, and the *Saudi Medical Journal*. The focus of this research was tied to just three keywords: traffic safety, driving, and road safety.

## Discussion

Research related to RTI and road traffic awareness first appeared outside of the GCC region in 1926 and 1966, respectively ^23^. Comparatively, the first publication related to RTI in the GCC region did not appear until 1981, confirming a lag in RTI research activity among GCC nations relative to other developed nations.

Following that first publication in 1981, RTI research productivity gradually increased in the GCC region but remained relatively low until 2015–2019, when the volume of RTI research substantially increased. A similar trend was reported in India; publication volumes related to road traffic injuries in India increased significantly after 2010 ^24^. These collective results suggest an increased recent focus on traffic safety in the GCC region by policymakers, planners, and academic researchers. This increased research productivity can also be attributed to an increased number of print and online publication journals, increased attention to this topic within the scientific community, increased networking and collaboration capabilities, and an increased focus from governmental and specialized agencies such as World Health Organization (WHO).

Disparities in RTI research productivity were observed between the GCC region and other developed nations as well as within the GCC region. The GCC nations with the highest number of publications included Saudi Arabia (126 publications), the UAE (84 publications), and Qatar (50 publications). This finding is consistent with results previously reported by Abdo *et al.* ^23^ who found that Saudi Arabia had the highest number of publications related to road traffic safety and road traffic awareness relative to all other GCC nations. Saudi Arabia has a higher population and more research institutions than other GCC nations.

Trends among publication volumes, citations, and impact were inconsistent. The 126 publications originating in Saudi Arabia were cited 890 times and had a citation impact of 7.06. Despite lower publication volumes (84 and 50 publications, respectively), publications originating in the UAE and Qatar had higher citation impact (7.79 and 7.76, respectively).

Regarding the publishing institutions, the UAE University had the highest RTI research productivity with 40 publications, which were cited 545 times with a citation impact of 13.64. King Saud University and Qatar University also had a high number of publications (37 and 29 publications, respectively).

Again, trends among publication volumes, citations, and impact were inconsistent. Although the Hamad Medical Corporation and the Al Ain Hospital had just 15 and 12 publications, respectively, the citation impact for these publications ranked highest (16.33 and 14.58, respectively). These results suggest that the most impactful RTI research publications are originating from medical institutions rather than from universities. Universities should therefore focus on improving the quality of their research to increase its impact.

Regarding individual authorship, only three authors in the GCC region had 10 or more RTI research publications: F.M. Abu-Zidan (17 publications), K. Shaaban (15 publications), and H.O. Eid (10 publications). The 17 publications by F.M. Abu-Zidan were cited 313 times with a citation impact of 18.41.

Similar to institutions, trends among publication volumes, citations, and citation impact among individual authors were inconsistent. K. Shaaban had a relatively high publication volume (15 publications) but a low number of citations (68 citations) and a low citation impact (4.53). Conversely, A.F. Hefny and M. Grivna had relatively low publication volumes (7 and 6 publications, respectively) but high numbers of citations (168 and 137 citations, respectively) and high citation impact (24.00 and 22.83, respectively).

Based on the bibliographic coupling analysis of RTI research publications originating in the GCC region, nations with the highest bibliographic coupling activity included Saudi Arabia (a 5,197 total link strength), the UAE (a 3,731 total link strength), and Qatar (a 3,290 total link strength). This finding is consistent with other RTI research productivity measures. The majority of academic RTI research originated in Saudi Arabia, the UAE, and Qatar. The largest proportion (23,448 publications) originated from Saudi universities. In terms of population size, Qatar was found to be performing well regionally, with a ratio of 1.6 publications to every 1,000 inhabitants. When compared with Western nations, however, RTI research productivity remains relatively low in the GCC region. Long-term transformational visions that emphasize quality education, such as Saudi Arabia’s VISION 2030, may help to bridge the gap between the GCC region and Western nations. The ground realities point to how difficult this task will be. For example, the number of Ph.D. students in Saudi Arabia per 1,000 inhabitants is 0.34 while the number of Ph.D. students in the United Kingdom per 1,000 inhabitants is 2.69.

Research, by its nature, is a collaborative process. In any bibliometric analysis, the level of research collaboration is an important index to assess the current status of research in a specific field. Considering research collaboration by nation reveals both the degree of international communication as well as the most influential nations in a particular research field. In this study, the highest level of collaboration occurred among Saudi Arabia and the United States (11 instances). This finding is consistent with a previous study’s findings that found that the highest numbers of collaborative publications in the field of Physical Sciences (1980–2014) were produced through partnerships in Saudi Arabia and the United States (23.31%) and Saudi Arabia and Egypt (22.95%) ^25^. Similarly, the highest numbers of collaborative publications related to Health Sciences in Saudi Arabia were produced through partnerships between Egypt (16.5%) and the United States (16.3%) ^26^. These collective findings highlight the strong collaborative and intellectual ties Saudi Arabia maintains with respective academic communities in the United States and Egypt. However, these ties should be further strengthened not only among the GCC nations but also with other developed countries such as Australia and Germany. Australia, the United States and Germany are currently leading the world in terms of best practices, regulations and road testing of connected and automated vehicles (CAVs) ^27^, which represent the future of transportation and traffic engineering and safety research.

We also considered the number of individual authors on each RTI research publication. Select publications had more than 10 individual authors, although this was relatively infrequent. Most RTI research publications originating in the GCC region had no more than five authors; 60 publications had a single author, 54 publications had two authors, and 74 publications had three authors. The trend depicted in Figure 6 shows a decline in publication collaborations involving four or more authors in the GCC region. Instead, publication collaborations involving a smaller number of authors has been favored. Also, the number of single author publications was high. Efforts to improve collaboration among individual authors from different departments and institutions would improve both the quality and impact of research originating in the GCC region.

Among the most prevalent publication journals for RTI research in the GCC region, *Accident Analysis and Prevention* had the highest number of publications (25), number of citations (533), and citation impact (21.32). Of the 20 most commonly cited RTI research publications originating in the GCC region, eight appeared in *Accident Analysis and Prevention*.

The most commonly cited RTI research publications originating in the GCC region related to traffic safety, and to a lesser extent, traffic awareness. A number of the commonly cited RTI research publications specifically addressed medical issues associated with these topics. This finding is consistent with findings previously reported by 24. Of the 20 most commonly cited RTI research publications originating in the GCC region ( Table 6), only two publications considered pedestrians (the most vulnerable road users): one published in *Accident Analysis and Prevention* ^21^ and one published in *Traffic Injury Prevention* ^28^. None of these publications considered more contemporary or emerging topics, such as the role of mobile phones in road traffic injuries.

Consistent with the publication-related findings, the most commonly listed keywords in RTI research publications originating in the GCC region were road traffic accidents (41 occurrences) and road traffic injuries (40 occurrences). Terms such as traffic safety (27 occurrences), road safety (20 occurrences), and road traffic crashes (19 occurrences) were also commonly listed. Terms such as mobile phones (5 occurrences), pedestrian safety (5 occurrences), pedestrians (5 occurrences), and distracted driving (4 occurrences) were least common.

To detect changes over time in RTI research focus in the GCC region, we considered topical trends from 1992 to 2018. In 1992–2007, RTI research publications were focused on the medical aspects of the traffic safety; hospital and mortality were common topics in RTI research publications. In 2008, the breadth of topics began to increase. In 2009–2019, the most common RTI research publication topics included road, traffic, safety, and accidents. This trend can be attributed to the increasing RTI burden in the GCC region, which experiences ever-increasing road traffic crashes and deaths on the roads each year despite the development of both in-vehicle and highway-based safety system technologies. Traffic safety awareness among governmental traffic planning department staff and academic researchers has increased due to improved international data collection and sharing practices and the periodic Global Status Reports on Road Safety in 2013 ^1^, 2015 ^5^, and 2018 ^29^ published by the WHO.

This aspect of the investigation not only revealed the historic RTI research focus in the GCC region, but also revealed important deficiencies that can guide future research. For example, public safety in the GCC region may benefit from future research focused on pedestrian behavior and safety or the effects of mobile phone usage and distracted driving.

## Future research directions

More broadly, future RTI research should focus on developing preventive actions to increase and promote traffic safety rather than only addressing the medical aspects of traffic injuries in the GCC region. This bibliometric analysis has revealed minimal research related to human behavior factors and pedestrians and cyclists who are most vulnerable for road traffic injuries in the GCC region. Traffic safety researchers and practitioners in the region should focus on these research areas, which show significant publication potential. Researchers should also focus on improving communications between departments and institutions to facilitate sharing and usage of updated data among researchers. This latter effort will also encourage collaboration among authors. As noted previously, efforts to improve collaboration among individual authors from different departments and institutions would improve both the quality and impact of research originating in the GCC region.

## Conclusions

To address the potential, disconnect between RTI research and any traffic safety mitigation measures put in place in the GCC region we performed a bibliometric analysis using publication data from the Web of Science database. We considered research productivity, institutional and individual authorship, bibliographic coupling and global collaboration, publication journals and publications, and keywords and associated topical trends for the GCC region. In addition, we performed a three-factor analysis using GCC nation, publication journal, and keyword parameters to obtain a more comprehensive understanding of RTI research in the GCC region.

The first publication related to road traffic injuries in the GCC region did not appear until 1981, confirming a lag in RTI research activity among GCC nations relative to other developed nations. More recently in 2015–2019, the volume of RTI research substantially increased, suggesting an increased focus on traffic safety in the GCC region. This increased focus was not uniform across the GCC region. Disparities in RTI research productivity were observed within the GCC region. The GCC nations with the highest and lowest publication volumes included Saudi Arabia and Bahrain respectively. Because most of the impactful RTI research publications originated from medical institutions rather than from universities, universities must focus on improving the quality of their research to increase its impact. Only three individual authors had 10 or more RTI research publications. Most RTI research publications originating in the GCC region had three collaborative authors, and to a lesser extent, a single author. Efforts to improve collaboration among individual authors from different departments and institutions would improve both the quality and impact of research originating in the GCC region.

Among the most prevalent publication journals for RTI research in the GCC region, *Accident Analysis and Prevention* had the highest number of publications, number of citations, and citation impact. Of the 20 most cited RTI research publications originating in the GCC region, four publications have been cited more than 50 times. Eight of the most cited RTI research publications appeared in *Accident Analysis and Prevention*. The most commonly listed keywords in RTI research publications were road traffic accidents and road traffic injuries. Terms such as mobile phones, pedestrian safety, pedestrians, and distracted driving were least common. This aspect of the investigation revealed the historic RTI research focus as well as important deficiencies that can guide future research in the GCC region.

Non-motorized transport users such as pedestrians and cyclists are the most neglected group in the road traffic research in GCC nation despite of having higher fatality rates. The researchers should focus on the safety impacts of the pedestrians and other non-motorized road users who are the most volatile and susceptible to road accidents. The researches related to road user behavior and on the usage of cell phones and seatbelts are also not on the satisfactory level in GCC. It is of utmost importance that the data sharing and collaboration between the institutes should be improved so that the quality and impact of the future RTI researches can be improved because of experienced team-work and professional feedbacks. This will also help to improve the current trend of researchers and institutions who are currently working in silos rather than in collaboration with the traffic practitioners, planners and professionals. 

Across several of the bibliometric parameters, trends among publication volumes, citations, and impact were inconsistent. For example, a high volume of publications did not always result in a high number of citations or a high citation impact.

This study demonstrated the merit of applying bibliometric analysis to guide decision-making related to RTI research. The collective results of this study indicated that—despite the recent increase in RTI research productivity in the GCC region—the quantity and quality of RTI research publications originating in the GCC nations is insufficient to meet the increasing burden of RTI in the region. More effort is needed to increase and promote traffic safety. The collective results from this study can inform governmental authorities and policy makers in the GCC region of research investment needs and support cost- effective resource allocation. In addition, the results from this study can inform academic researchers of research deficiencies and impactful publishing venues. An informed and collective focus on RTI research in the GCC region will help to ensure broader public safety.

## Data availability

Figshare: Bibliometric analysis of road traffic injuries research in the Gulf Cooperation Council region

https://doi.org/10.6084/m9.figshare.12855572 ^16^

This project contains the following underlying data:

- 312 Publications Record CSV.csv. (Results of all 312 publication records used in this study)
- RTI_Gulf22Dec19.xlsx. (Excel data sheets and Charts data)

Data are available under the terms of the Creative Commons Zero "No rights reserved" data waiver (CC0 1.0 Public domain dedication).
