# Supplementary material for: Bibliometric analysis of road traffic injuries research in the Gulf Cooperation Council region
Source: F1000Res. 2020 Oct 16;9:1155. Originally published 2020 Sep 18. [Version 2] doi: 10.12688/f1000research.25903.2 (PMC7542249; doi:10.12688/f1000research.25903.2)
Supplement: Peer review history

# Peer review: Reviewer response for version 2

**DOI:** 10.5256/f1000research.30131.r73283

**Published:** 2020-11-16

**Authors:** Ashar Ahmed

**Affiliations:** 1 Department of Urban and Infrastructure Engineering, NED University of Engineering and Technology, Karachi, Pakistan

No new comments.

Is the work clearly and accurately presented and does it cite the current literature?

Yes

If applicable, is the statistical analysis and its interpretation appropriate?

Not applicable

Are all the source data underlying the results available to ensure full reproducibility?

Yes

Is the study design appropriate and is the work technically sound?

Yes

Are the conclusions drawn adequately supported by the results?

Yes

Are sufficient details of methods and analysis provided to allow replication by others?

Yes

Reviewer Expertise:

Road Safety Modelling, Accident Investigation

I confirm that I have read this submission and believe that I have an appropriate level of expertise to confirm that it is of an acceptable scientific standard.

# Peer review: Reviewer response for version 2

**DOI:** 10.5256/f1000research.30131.r73285

**Published:** 2020-10-19

**Authors:** Elhashemi M. Ali

**Affiliations:** 1 Department of Public Works, Civil Engineering, Faculty of Engineering, Cairo University, Cairo, Egypt

No further comments to make

Is the work clearly and accurately presented and does it cite the current literature?

Yes

If applicable, is the statistical analysis and its interpretation appropriate?

Yes

Are all the source data underlying the results available to ensure full reproducibility?

Yes

Is the study design appropriate and is the work technically sound?

Yes

Are the conclusions drawn adequately supported by the results?

Yes

Are sufficient details of methods and analysis provided to allow replication by others?

Yes

Reviewer Expertise:

Traffic Safety, Naturalistic Driving Studies, Big Transportation Data Analysis.

I confirm that I have read this submission and believe that I have an appropriate level of expertise to confirm that it is of an acceptable scientific standard.

# Peer review: Reviewer response for version 2

**DOI:** 10.5256/f1000research.30131.r73282

**Published:** 2020-10-19

**Authors:** Uneb Gazder

**Affiliations:** 1 Department of Civil Engineering, University of Bahrain, Zallaq, Bahrain

I am satisfied with the changes being made in response to my comments.

Is the work clearly and accurately presented and does it cite the current literature?

Yes

If applicable, is the statistical analysis and its interpretation appropriate?

Not applicable

Are all the source data underlying the results available to ensure full reproducibility?

Yes

Is the study design appropriate and is the work technically sound?

Yes

Are the conclusions drawn adequately supported by the results?

Partly

Are sufficient details of methods and analysis provided to allow replication by others?

Yes

Reviewer Expertise:

Traffic engineering

I confirm that I have read this submission and believe that I have an appropriate level of expertise to confirm that it is of an acceptable scientific standard.

# Peer review: Reviewer response for version 1

**DOI:** 10.5256/f1000research.28586.r71721

**Published:** 2020-10-09

**Authors:** Muhammad Adnan

**Affiliations:** 1 Transportation research institute (IMOB), Hasselt University, Diepenbeek, Belgium

This is a nice effort from the authors, however, I see that some of the interpretations and conclusions of the results are not appropriate. Please find my specific comments below: 

 Introduction: 

 Para 4, line 3: Historical RTI research....to traffic crashes. What do you mean here by historical RTI research.. Sentence seems a bit confusing.

 Para 5, line 5: Unlike demonstrated efforts....put in place. Could you please explain here what were those demonstrations and how you have concluded here that there is a disconnection in GCC. 

 Results: 

 Sub-section : Three factor analysis...

- Why 3 factors are considered?
- I see the importance of nations and keywords but not able to comprehend inclusion of journal (what exactly it will reflect it is not clear to me).

Discussion: 

 Para 1: Could you please discuss here what was the reason why before 1981 there wasn't any research study related to RTI? Is it because the RTI problem was not that significant or was no attention given by academics or no funding was available or there wasn't any research culture or academic performance was not graded based on research activity...which is clearly the case now. 

 Para 4: why articles from KSA have low impact factor compared to UAE and Qatar? Could you elaborate on the possible reasons for this?

 Para 6: medical journals usually have way higher impact factors and have large audience compared to non-medical journals (planning and engineering-based journals). I do not agree on the conclusion you made here that universities are not producing high-quality research just based on the citations impact. 

 Para 8: My comment here is similar to what I mentioned above, authors need to categories, you cannot ignore the content and make your conclusion on citations.

 Para 12, Line 1: Select publications had... The sentence should start with …Selected...

Is the work clearly and accurately presented and does it cite the current literature?

Yes

If applicable, is the statistical analysis and its interpretation appropriate?

Partly

Are all the source data underlying the results available to ensure full reproducibility?

Yes

Is the study design appropriate and is the work technically sound?

Partly

Are the conclusions drawn adequately supported by the results?

Partly

Are sufficient details of methods and analysis provided to allow replication by others?

Yes

Reviewer Expertise:

Transport Planning, Road Safety, Transport Modelling, Big data.

I confirm that I have read this submission and believe that I have an appropriate level of expertise to confirm that it is of an acceptable scientific standard, however I have significant reservations, as outlined above.

# Peer review: Reviewer response for version 1

**DOI:** 10.5256/f1000research.28586.r71722

**Published:** 2020-10-07

**Authors:** Ashar Ahmed

**Affiliations:** 1 Department of Urban and Infrastructure Engineering, NED University of Engineering and Technology, Karachi, Pakistan

This paper provides a review of the Road Traffic Incident (RTI) research publication frequency across Gulf Cooperation Council region. The country with the highest RTI research productivity, most productive institution for RTI research, most productive author, most prevalent journal for RTI publication, topics of RTI research over the period of time, and future research directions in the GCC region were explored. It was found that the majority of the research was related to medical aspects then engineering aspects of RTI. The findings of this study are very critical which makes the paper extremely worthy of publication.

 The following comments will improve the manuscript:

- The authors claim that RTIs are the 9 ^th^ leading cause of deaths. As per the WHO Global Status Report on Road Safety 2018, RTIs are the 8 ^th^ leading cause of deaths around the world. This may be updated in the ‘Introduction’ section.
- In Table 5 the word ‘behavior’ is in a separate row. It is part of the name of the journal and should be in the preceding row.

Is the work clearly and accurately presented and does it cite the current literature?

Yes

If applicable, is the statistical analysis and its interpretation appropriate?

Not applicable

Are all the source data underlying the results available to ensure full reproducibility?

Yes

Is the study design appropriate and is the work technically sound?

Yes

Are the conclusions drawn adequately supported by the results?

Yes

Are sufficient details of methods and analysis provided to allow replication by others?

Yes

Reviewer Expertise:

Road Safety Modelling, Accident Investigation

I confirm that I have read this submission and believe that I have an appropriate level of expertise to confirm that it is of an acceptable scientific standard.

# Response

**Published:** 2020-10-07

**Authors:** Shafiq Ur Rehman

**Affiliations:** Imam Abdulrahman Bin Faisal University, Saudi Arabia

1. This is updated in the Introduction section (line 5).

2. It is corrected in Table 5.

# Peer review: Reviewer response for version 1

**DOI:** 10.5256/f1000research.28586.r71716

**Published:** 2020-09-28

**Authors:** Uneb Gazder

**Affiliations:** 1 Department of Civil Engineering, University of Bahrain, Zallaq, Bahrain

This paper presents a comprehensive bibliographic analysis of the research related to road safety in GCC countries. All important trends have been analyzed in detail and their possible reasons have been discussed. The authors have also highlighted important issues related to past trends and suggested future improvements. The following corrections are required to be made in the manuscript before accepting it for indexing:

- Figure 1 is not cited before its appearance.
- Data in table 1 should be updated.
- There are repetitions in the discussion section, for e.g., an explanation of bibliographic coupling and collaboration that have already been done in the previous sections. They should be removed from the discussion.
- Limitations should be included in the introduction or method section.
- The authors have mentioned important findings in the discussion related to research trends and their potential future directions (such as pedestrian accidents). These points should be reiterated in the conclusion. Details about the number of publications, authors, etc. can be omitted or reduced in conclusions.

Is the work clearly and accurately presented and does it cite the current literature?

Yes

If applicable, is the statistical analysis and its interpretation appropriate?

Not applicable

Are all the source data underlying the results available to ensure full reproducibility?

Yes

Is the study design appropriate and is the work technically sound?

Yes

Are the conclusions drawn adequately supported by the results?

Partly

Are sufficient details of methods and analysis provided to allow replication by others?

Yes

Reviewer Expertise:

Traffic engineering

I confirm that I have read this submission and believe that I have an appropriate level of expertise to confirm that it is of an acceptable scientific standard, however I have significant reservations, as outlined above.

# Response

**Published:** 2020-10-07

**Authors:** Shafiq Ur Rehman

**Affiliations:** Imam Abdulrahman Bin Faisal University, Saudi Arabia

1. Figure 1 is already cited in the text before its appearance. Kindly refer to the Introduction section First paragraph (Lines 3-4).

 2. The data used in table 1 is per the Statistical survey of 2013 and reported in the Global Status Report on Road Safety 2015 published by WHO in the Country Profiles (pages 77-255).

 The reason for not using the WHO 2018 report is that the data for Kingdom of Bahrain is not available in it and for the consistent comparison with other GCC countries it could not be used.

 Reports links:

Reference Source (2015)

Reference Source (2018)

 3. The mentioned paragraphs have been removed from the discussions section (Lines 309-318).

 4. We have moved the limitations in the method section

 5. The findings are reiterated in the conclusions (Lines 454-464).

 Details about the number of publications, authors are also reduced from the conclusions.

# Peer review: Reviewer response for version 1

**DOI:** 10.5256/f1000research.28586.r71718

**Published:** 2020-09-22

**Authors:** Elhashemi M. Ali

**Affiliations:** 1 Department of Public Works, Civil Engineering, Faculty of Engineering, Cairo University, Cairo, Egypt

This article has a great sound of knowledge since it summarized many research efforts to obtain a comprehensive understanding of Road Traffic Injuries (RTI) and presented them in a Bibliometric analysis. The article flow is easy to follow and it was written well.

 The reviewer has some comments that might add to the paper and these comments are as follows:

1. In the method section: can the authors present the TS equation in is a flow chart, a figure, or a table instead of this long equation of research terms?
2. The sentence "The authors did not apply any filter related to languages and time period to achieve a comprehensive view of RTI research" need to be revised as there is already a time period between 1981 and 2019 that the article focuses on.
3. The "three-factor analysis" need to be introduced and explained in details.

Is the work clearly and accurately presented and does it cite the current literature?

Yes

If applicable, is the statistical analysis and its interpretation appropriate?

Yes

Are all the source data underlying the results available to ensure full reproducibility?

Yes

Is the study design appropriate and is the work technically sound?

Yes

Are the conclusions drawn adequately supported by the results?

Yes

Are sufficient details of methods and analysis provided to allow replication by others?

Yes

Reviewer Expertise:

Traffic Safety, Naturalistic Driving Studies, Big Transportation Data Analysis.

I confirm that I have read this submission and believe that I have an appropriate level of expertise to confirm that it is of an acceptable scientific standard.

# Response

**Published:** 2020-10-07

**Authors:** Shafiq Ur Rehman

**Affiliations:** Imam Abdulrahman Bin Faisal University, Saudi Arabia

1. The flow chart has been added (see figure 2)

2. We did not apply any filter of time span while searching our query. The year 1981 was the date of first publication of the topic and 2019 was last publication as a result of our search query.

3. The detail about three factor analysis has been added.
